# Correlating Oxygen Evolution Catalysts Activity and Electronic Structure by a High-Throughput Investigation of Ni_1-y-z_Fe_y_Cr_z_O_x_

**DOI:** 10.1038/srep44192

**Published:** 2017-03-13

**Authors:** Christoph Schwanke, Helge Sören Stein, Lifei Xi, Kirill Sliozberg, Wolfgang Schuhmann, Alfred Ludwig, Kathrin M. Lange

**Affiliations:** 1 Operando Characterization of Solar Fuel Materials, Helmholtz-Zentrum Berlin für Materialien und Energie, Albert-Einstein-Str. 15, 12489 Berlin, Germany; 2 Chair for MEMS Materials, Institute for Materials, Ruhr-University Bochum, Universitätsstr. 150, 44801 Bochum, Germany; 3 Analytical Chemistry - Center for Electrochemical Sciences (CES), Ruhr-University Bochum, Universitätsstr. 150, 44780 BochumGermany; 4 Materials Research Department, Ruhr-University Bochum, Universitätsstr. 150, 44801 Bochum Germany; 5 Universität Bielefeld, Physikalische Chemie, Universitätsstr. 25, D-33615 Bielefeld, Germany

## Abstract

High-throughput characterization by soft X-ray absorption spectroscopy (XAS) and electrochemical characterization is used to establish a correlation between electronic structure and catalytic activity of oxygen evolution reaction (OER) catalysts. As a model system a quasi-ternary materials library of Ni_1-y-z_Fe_y_Cr_z_O_x_ was synthesized by combinatorial reactive magnetron sputtering, characterized by XAS, and an automated scanning droplet cell. The presence of Cr was found to increase the OER activity in the investigated compositional range. The electronic structure of Ni^II^ and Cr^III^ remains unchanged over the investigated composition spread. At the Fe L-edge a linear combination of two spectra was observed. These spectra were assigned to Fe^III^ in O_h_ symmetry and Fe^III^ in T_d_ symmetry. The ratio of Fe^III^ O_h_ to Fe^III^ T_d_ increases with the amount of Cr and a correlation between the presence of the Fe^III^ O_h_ and a high OER activity is found.

To acquire an in-depth understanding of specific catalysts’ properties advanced analytical techniques are needed that can correlate electronic structure to functional properties. This may lead to the formulation of generic pathways and design criteria for highly complex reactions such as the oxygen evolution reaction (OER). One of such analysis techniques is X-ray absorption spectroscopy (XAS) that directly probes the unoccupied electronic structure. Making this technique accessible for combinatorial materials science allows for the correlation of electronic structure, crystallographic data and electrochemical performance and therefore expedites materials design. In the present work, a first attempt is presented to correlate electronic structure and electrochemical properties probed by high-throughput transition metal L-edges XAS and a scanning droplet cell (SDC) on a Ni-Fe-Cr-O continuous composition spread combinatorial materials library for the OER. The system Ni_1-y-z_Fe_y_Cr_z_O_x_ was chosen, because Ni-Fe oxides^1^,^2^,^3^,^4^ are relevant OER catalysts and it was shown that the alloying of Cr into the spinel increased the electrocatalytic activity of Ni_1-y_Fe_y_O_4_^5^.

Complementary to the here in proposed experimental route of gaining a mechanistic understanding, high-throughput computational studies^6^,^7^,^8^,^9^ aiming at theory driven discovery were presented in literature. An important example for computationally guided design is found for perovskite based OER catalysts. For perovskite OER catalysts a generally accepted design principle is based on the occupancy of the e_g_ (σ) orbital close to unity if the ligand environment of the transition metal shows octahedral (O_h_) symmetry^10^,^11^,^12^. Other factors such as covalence, 3d occupation and geometry, however, are important as well^13^. The reasoning behind the single occupancy of the e_g_ orbital is, that in this case the bonding strength between transition metal and absorbed oxygen species is neither too strong (low e_g_ occupancy) nor too weak (high e_g_ occupancy) and thus the *Sabatier principle* is fulfilled.

The correlation of such fundamental changes in electronic structure and their subtle influence on electrochemical properties, is however already challenging to elucidate for quasi-binary oxides. Whereas until very recently it was assumed for Ni-Fe-O catalysts that Ni was the catalytically active site^14^, recent DFT studies supported by XAS data suggest that Fe is the active center for the observed OER activity^15^. For Ni_1-y-z_Fe_y_Cr_z_O_x_, which is the focus of this work, Singh *et al*. revealed by XPS that the surface of the film contains Ni^2+^, Fe^3+^, Cr^3+^ and Cr^6+^ and found an improvement of OER with the incorporation of Cr. They tentatively assigned the hexavalent Cr-ion as OER active center, but the exact role of Cr is not well understood^5^.

For a thorough understanding of the functioning of OER catalysts direct probing of the involved 3d orbitals is performed by XAS at the transition metal L-edges. Combined with high-throughput electrochemical measurements an experimental study on the correlation of electronic structure and electrochemistry of Ni_1-y-z_Fe_y_Cr_z_O_x_ based OER catalyst is presented. In this study 130 elemental compositions of Ni_1-y-z_Fe_y_Cr_z_O_x_ were probed by XAS at all three metal L-edges and subsequently investigated for their electrochemical activity.

## Results

**Figure 1:**
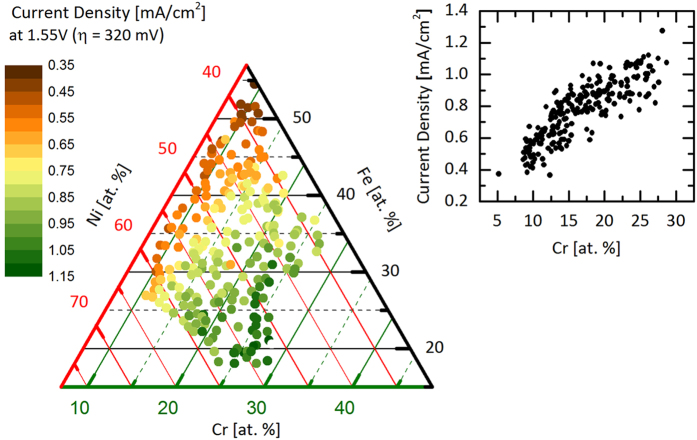
Color coded ternary plot of the composition spread of the Ni_1-y-z_Fe_y_Cr_z_O_x_ materials library showing the current density at 1.55 V corresponding to a thermodynamic overpotential of η = 320 mV. The current increases almost linearly with Cr content as shown in the inset.

The materials library of Ni_1-y-z_Fe_y_Cr_z_O_x_ was centered around the composition 50 at. % Ni, 25 at. % Fe and 25 at. % Cr omitting the oxygen content as shown in the ternary composition spread in Fig. 1. Unless stated otherwise, the elemental compositions are reported without oxygen as it could not reliably be determined using energy dispersive X-ray spectroscopy (EDX). The obtained compositional spread was 10–30 at. % Cr, 20–55 at. % Fe, and 40–65 at. % Ni.

### Electrochemical evaluation

To evaluate the electrochemical activity for the OER in correlation with XAS the materials library was investigated by an automated SDC. The corresponding Tafel slopes (surface area independent) and current densities are reported.

In Fig. 1 the current density at an overpotential of *η* = 320 mV (1.55 V vs. reversible hydrogen electrode) is shown. At this potential the oxygen evolution rate is low and hence the current density is solely determined by the OER kinetics. A clear trend to higher current densities with higher Cr content is observed. The current densities obtained at these relatively low overpotentials, even with assumed flat surfaces, are relevant for the application in solar water splitting where current densities between 1–10 mA/cm^2^ are commonly achieved. For practical applications the current density can be increased by increasing the electrochemical active surface area, e.g. by nanostructuring. In order to exclude that the increase of current density that we observed is caused by changes in the surface area the sample was investigated with atomic force microscopy (AFM). AFM images were recorded in total at ten different positions of the materials library. These positions were systematically chosen to cover the full range of the investigated material compositions in order to test for material composition dependent changes in morphology. In Figure S1 in the Supplementary Information AFM measurements of the Ni_1-y-z_Fe_y_Cr_z_O_x_ materials library at compositions with low Cr content (50 at. % Ni, 35 at. % Fe, 15 at. % Cr) and high Cr content (48 at. % Ni, 12 at. % Fe, 40 at. % Cr) are shown as examples. The investigated surfaces have the same electrochemically active surface area within the error range. (rms-roughness: 37 ± 1 nm, ratio of microscopic to geometric surface area: 1.228 ± 0.002).

**Figure 2:**
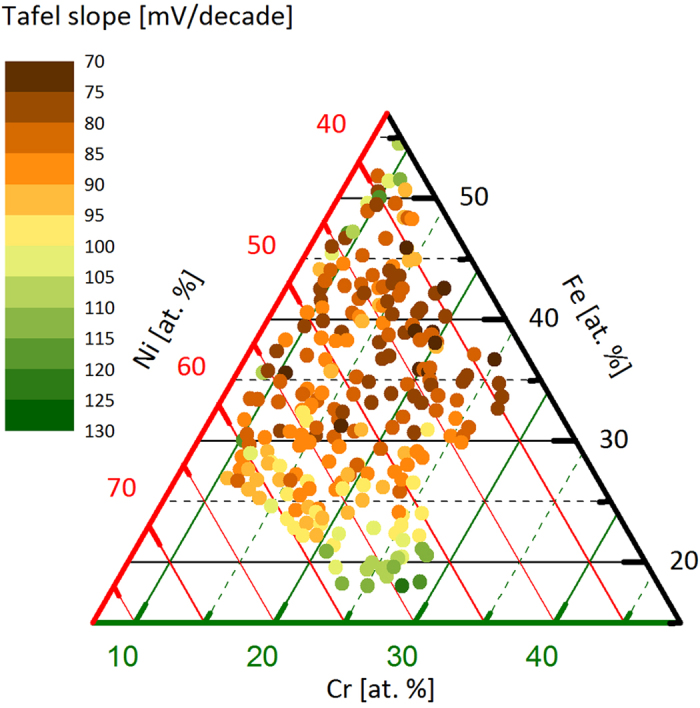
Color coded ternary plot of the composition spread of the Ni_1-y-z_Fe_y_Cr_z_O_x_ materials library showing the Tafel slope. The slope was determined in the voltage region of 1.50 V to 1.54 V.

The Tafel slope is shown in Fig. 2. The observed Tafel slopes are in the range from 70 to 115 mV/decade, which is rather high as compared to 60 mV/decade observed for Co-perovskites^16^ or amorphous Co-oxides^17^,^18^ and 40 mV/decade measured for Ni/Fe-oxides^5^,^14^,^19^,^20^. The lower thermodynamic limits in the Butler-Volmer formalism predict Tafel slopes of 24, 40 or 60 mV/decade^21^. It should be noted that in a scanning droplet cell slightly different conditions as compared with rotating disk electrode experiment exist, meaning that the reported Tafel slopes are rather an upper boundary. Since the Tafel slope is relatively constant above 30% Fe, it is probable that the rate-determining step (RDS) stays the same in this region. Below 30% Fe the Tafel slope increases from around 70 mV/decade to up to 115 mV/decade.

### XAS at the transition metal L-edges

**Figure 3:**
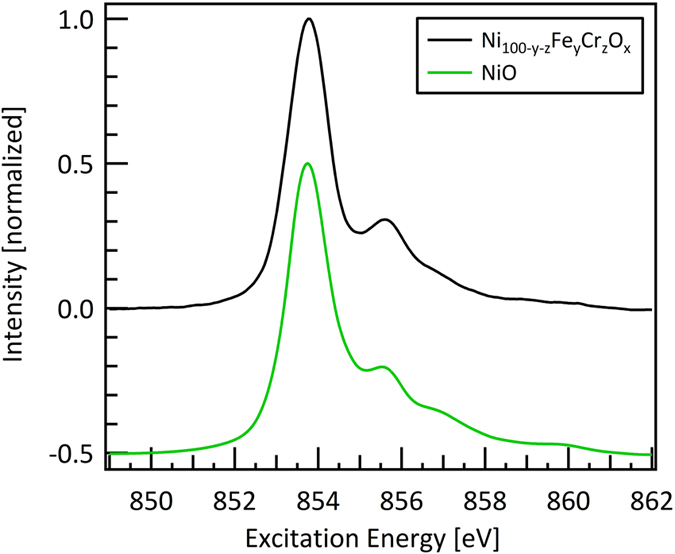
Ni L_3_-edge of Ni_1-y-z_Fe_y_Cr_z_O_x_. The Ni L_3_-edge was found to be constant across the entire materials library. The NiO reference spectrum is adopted from^22^ and shifted downwards by 0.5 for better readability.

**Figure 4:**
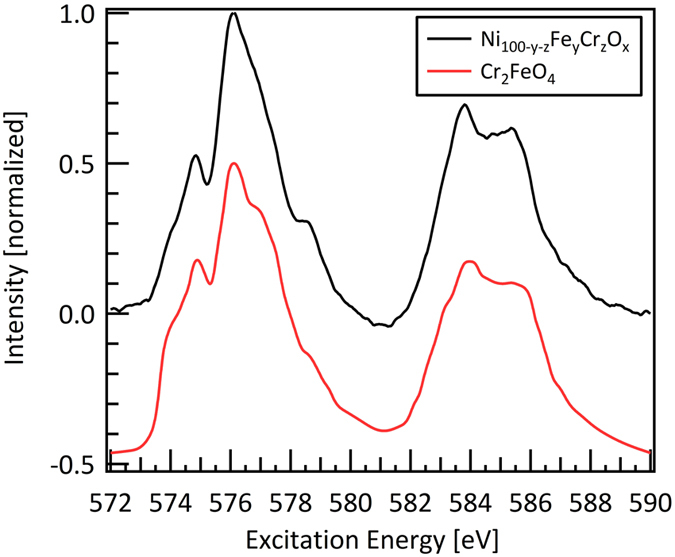
Cr L_3_- and Cr L_2_-edge of Ni_1-y-z_Fe_y_Cr_z_O_x_. No variation of the Cr L-edges is observed across the entire materials library. The reference spectrum of Cr_2_FeO_4_ is adopted from^23^ and shifted downwards by 0.5 for better readability.

To gain insight into the changes of the electronic structure of the involved transition metals, the transition metals L-edges were evaluated across the materials library. No changes were found across the investigated elemental compositions in the L-edge spectra of Ni and Cr, whereas for Fe drastic changes occurred. A characteristic spectrum of the Ni L-edge is shown in Fig. 3. This spectrum is in very good agreement with the NiO reference spectrum^22^ shown in the same Fig., which indicates that Ni prevails as Ni^II^ in the materials library. A characteristic Cr spectrum of the measurement series is shown in Fig. 4. Based on the Cr_2_FeO_4_ reference spectrum^23^ it was identified as a Cr^III^ species.

**Figure 5:**
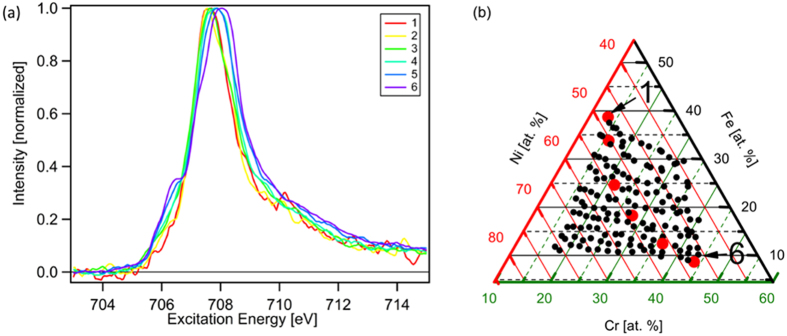
(**a**) Fe L_3_-edge of Ni_1-y-z_Fe_y_Cr_z_O_x_ of the compositions 1–6 denoted in the ternary diagram in (**b**).

**Figure 6:**
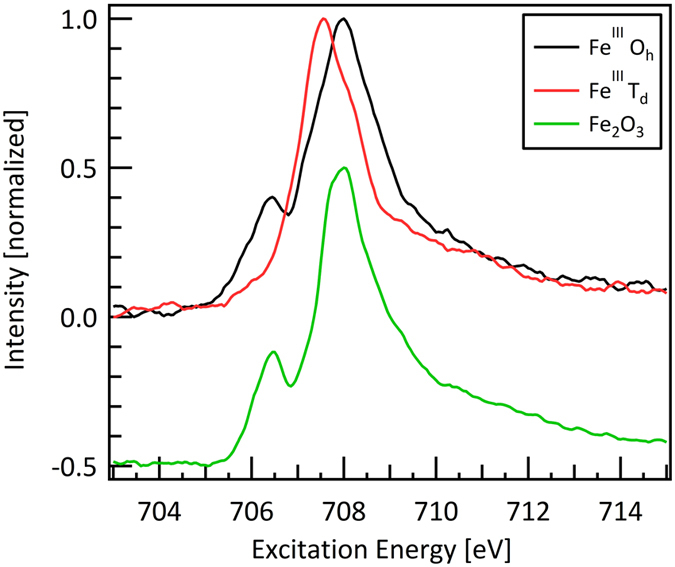
Fe L_3_-edge of Fe^III^ O_h_ and Fe^III^ T_d_ species. The two spectra are linear components of the Fe L_3_-edge spectra of Ni_1-y-z_Fe_y_Cr_z_O_x_ given in Fig. S2 in the Supplementary Information. The Fe_2_O_3_ reference spectrum is taken from^22^ and shifted downwards by 0.5 for better readability.

Examples of Fe L-edge spectra are shown in Fig. 5. Fe is present in the Fe^III^ state as deduced from the energy positions of the L-edge spectra^24^. Two linear components were determined from the dataset of Fe L-edges as shown in Fig. 6. The first component is very similar to the spectrum of Fe_2_O_3_ in accordance with Fe^III^ in octahedral symmetry, thus we denote this spectrum as Fe^III^ O_h_. The second component is similar to spectra of the compound FePO_4_ in which Fe^III^ is in tetrahedral coordination^25^ and this spectrum is denoted as Fe^III^ T_d_. A small energy shift was found between the Fe L-edge of FePO_4_ obtained from literature and the observed Fe^III^_B_ species. The presence of Fe^III^ in D_4h_ symmetry was excluded by comparison to multiplet simulations shown in Fig. S2 in the Supplementary Information.

**Figure 7:**
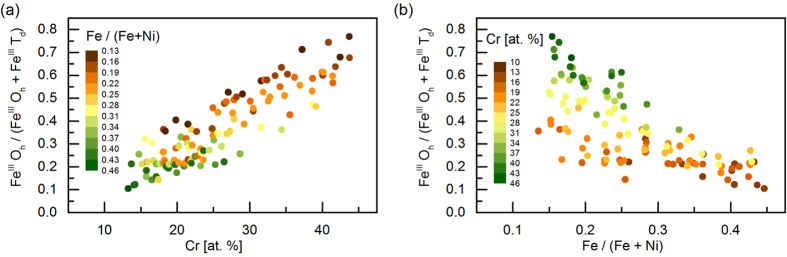
Ratio of the amounts of the two Fe species Fe^III^ O_h_ and Fe^III^ T_d_ plotted over (**a**) the Cr content, (**b**) Fe/(Fe + Ni) content as measured by EDX. Spectra for these species are shown in Fig. 6. The color code denotes the compositional ratio of Fe/(Fe + Ni). There is a stronger correlation of the ratio Fe^III^ O_h_/(Fe^III^ O_h_ + Fe^III^ T_d_) to the Cr content than to the ratio Fe/(Fe + Ni).

Linear combination fitting was performed on the whole dataset of Fe L-edges with the two spectra Fe^III^ O_h_ and Fe^III^ T_d_. No systematic trend of the error of the fit was found (see Fig. S3 in the Supplementary Information), which confirms that indeed the chosen references are adequate for the fit and no third component is present. The thus obtained amounts of the species Fe^III^ O_h_ and Fe^III^ T_d_ are shown in Fig. 7 and as ternary plot as Fig. S4 in the Supplementary Information. It is found that the amount of the Fe^III^ O_h_ species increases strongly with the Cr content. Also, it decreases weakly with the ratio Fe/(Ni + Fe) for a constant amount of Cr.

## Discussion

Ni was found to be in the Ni^II^ state and no clear increase of the OER activity with the Ni content was observed. It was previously shown^9^ that NiO (containing exclusively Ni^II^ species) exhibited a relatively high overpotential as compared to LaFeO_3_ and SrFeO_3_ suggesting that Ni^II^ is not forming the active center for OER catalysis.

The increase of OER activity with Cr content, while only Cr^III^ is present, is either caused by Cr being the active species or a positive effect on Fe or Ni and one of those being the active species. We consider it unlikely that Cr^III^ is the active site, since six 3d electrons as found in Cr^III^, result in a too strong Cr-O bond and hence should lead to a low OER activity. The situation may be different in Cr^VI^, but different from Singh *et al*.^5^ we did not observe the presence of Cr^VI^.

As shown in Figs 2 and 7, the ratio Fe^III^ O_h_/(Fe^III^ T_d_ + Fe^III^ O_h_) increases with the amount of Cr, similarly to the current density at an overpotential of 320 mV. This either indicates that the presence of Fe^III^ T_d_ has a negative effect on the OER activity or that Fe^III^ O_h_ has a positive effect, or both.

We exclude Fe^III^ T_d_ being the active site for two reasons, namely the correlation between the amount of Fe^III^ T_d_ and the OER activity and the electronic structure of Fe^III^ in a T_d_ symmetric ligand environment (LS), which is 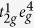. The high e_g_ electron count results in a too weak Fe-O bond, making Fe^III^ T_d_ supposedly relatively inactive for the OER.

Since the observed OER current increases with Fe^III^ O_h_ this species is a candidate for being involved in the active site. Also the electronic configuration of Fe^III^ O_h_ (LS), 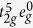, is very different from the Fe^III^ T_d_ case. One example is the semiconductor Fe_2_O_3_, which is often used in solar water splitting despite its very low charge carrier lifetime. It is then usually combined with an OER catalysts, due to its relatively low OER activity^26^,^27^.

## Conclusion

In the material system Ni_1-y-z_Fe_y_Cr_z_O_x_ an increase in OER activity with the introduction of Cr was observed using a SDC. Tafel slopes were found to be in the range of 70 to 115 mV/decade with a relatively constant slope of 80 mV/decade for Fe concentrations above 30%. This increase in activity was investigated by XAS of the transition metal L-edges. Ni and Cr were found to be in the Ni^II^ and Cr^III^ states throughout the whole composition range with electronic structures similar to NiO and Cr_2_FeO_4_. Fe on the contrary showed variations with the elemental compositions, which were assigned to two species Fe^III^ O_h_ and Fe^III^ T_d_. The amount of Fe^III^ O_h_ relative to the total amount of Fe was found to increase with Cr content. Since the OER activity also increases with the amount of Cr, either Fe^III^ T_d_ has a negative effect on the OER activity or Fe^III^ O_h_ has a positive effect.

## Methods

### Sample Preparation

Thin film materials libraries were deposited in a combinatorial reactive magnetron sputtering system (AJA International ATC-2200 V) as described in ref. 28. The substrate used was a 7.5 × 7.5 cm FTO coated glass substrate. The pressure prior to sputtering was below 0.66·10^−8^ Pa. The reactive atmosphere of Ar/O_2_ was kept at a constant pressure of 0.66 Pa over the entire deposition with an Ar flow of 6.66·10^−7^ m^3^s^−1^ and an O_2_ (6 N purity) flow of 8.33·10^−8^ m^3^s^−1^. In the cylindrical deposition chamber the azimuthal angle was 90°, with the Ni and Fe targets at opposite sides and the Cr target in between them. The deposition of Fe was performed by direct current (DC) magnetron sputtering with a power of 30 W. Cr was sputtered with a radio frequency (RF) source at 280 W. Ni was sputtered using a pulsed direct current (pDC) power source run at 65 W. No intentional substrate heating was performed during deposition. After deposition the materials library was annealed in air at 873 K for 90 min with a heating rate of 16 K/min and a cooling rate of 4 K/min. Two materials libraries were deposited subsequently, one for electrochemical measurements and one for XAS. Elemental composition was determined by EDX using 20 kV acceleration voltage and an INCA x-act detector from Oxford mounted to a Jeol 5800 SEM.

### Electrochemical Measurements

The electrochemical high-throughput characterization of one materials library was performed using an automated SDC as described in refs 29, 30, 31. The SDC contained a double junction reference electrode (Ag/AgCl/3M KCl/0.1M KOH) with a potential of +239 mV vs. the normal hydrogen electrode (NHE) and a Pt-wire counter electrode housed in a special PTFE tip that was mounted on a three-axes micropositioning system^32^. Prior to the electrochemical measurements an electrochemical impedance spectrum (EIS) was acquired. Using this EIS the cell resistance was determined to perform a correction of the ohmic drop. To account for possible instability due to the electrochemical measurements two conditioning curves were performed prior to the actual electrochemical measurement under the exact same conditions. From this there is no obvious corrosion of the sample visible before and after the electrochemical measurement. The potentials were swept at a scan rate of 10 mV/s. All electrochemical measurements were performed in an aqueous solution of oxygen saturated 0.1M KOH. Both half-waves of cyclic voltammogramms were averaged, which allowed minimization of the contribution of capacitive currents. From these mean currents the tafel slopes were determined by fitting a line to the logarithm of the mean current density in the voltage range from 1.50 V to 1.54 V. In this potential range good linearity was observed. All potentials are reported in V vs. the reversible hydrogen electrode (RHE). It should be noted, that there are alternative ways to determine the tafel slope as for example by rotating disk electrodes than the one employed here. It is however believed that the comparability between the tafel slopes presented herein is good since all experiments are performed robotically under the exact same conditions.

### X-ray Absorption Spectroscopy

XAS measurements were performed with the LiXEdrom 2.0 endstation at the soft X-ray beamline UE56/2-PGM2 at BESSY II. An automated measurement system was set up to measure over 300 XAS spectra of the Ni, Fe and Cr L-edges in total fluorescence yield mode. Three GaAs photodiodes (Hamamatsu G1127) were employed as detectors. No radiation damage was observed in repeated measurements on the same position on the sample. Spectra were normalized to maximum intensity and a linear background subtraction was performed. The L-edges of Ni, Fe and Cr were energy calibrated with references of NiO^22^, Fe_2_O_3_^22^ and Cr_2_FeO_4_^23^.

## Additional Information

**How to cite this article:** Schwanke, C. *et al*. Correlating Oxygen Evolution Catalysts Activity and Electronic Structure by a High-Throughput Investigation of Ni_1-y-z_Fe_y_Cr_z_O_x_. *Sci. Rep.* **7**, 44192; doi: 10.1038/srep44192 (2017).

**Publisher's note:** Springer Nature remains neutral with regard to jurisdictional claims in published maps and institutional affiliations.

## Supplementary Material

Supplementary Information
