# Supplementary material for: Correlating Oxygen Evolution Catalysts Activity and Electronic Structure by a High-Throughput Investigation of Ni1-y-zFeyCrzOx
Source: Sci Rep. 2017 Mar 13;7:44192. doi: 10.1038/srep44192 (PMC5347035; doi:10.1038/srep44192)
Supplement: Supplementary Information [file srep44192-s1.pdf]

Supplementary Information

Correlating Oxygen Evolution Catalysts Activity and Electronic Structure by a High-Throughput Investigation of $Ni_{1-y-z}Fe_yCr_zO_x$

Christoph Schwanke[1+], Helge Sören Stein[2+], Lifei Xi[1], Kirill Sliozberg[3], Wolfgang Schuhmann[3,4], Alfred Ludwig[2,4] and Kathrin M. Lange[1]*

(1) Operando Characterization of Solar Fuel Materials, Helmholtz-Zentrum Berlin für Materialien und Energie, Albert-Einstein-Str. 15, 12489 Berlin, Germany

(2) Chair for MEMS Materials, Institute for Materials, Ruhr-University Bochum, Universitätsstr. 150, 44801 Bochum, Germany

(3) Analytical Chemistry - Center for Electrochemical Sciences (CES), Ruhr-University Bochum, Universitätsstr. 150, 44780 Bochum Germany

(4) Materials Research Department, Ruhr-University Bochum, Universitätsstr. 150, 44801 Bochum Germany

[+]these authors contributed equally to this work

[*]Kathrin.Lange@helmholtz-berlin.de

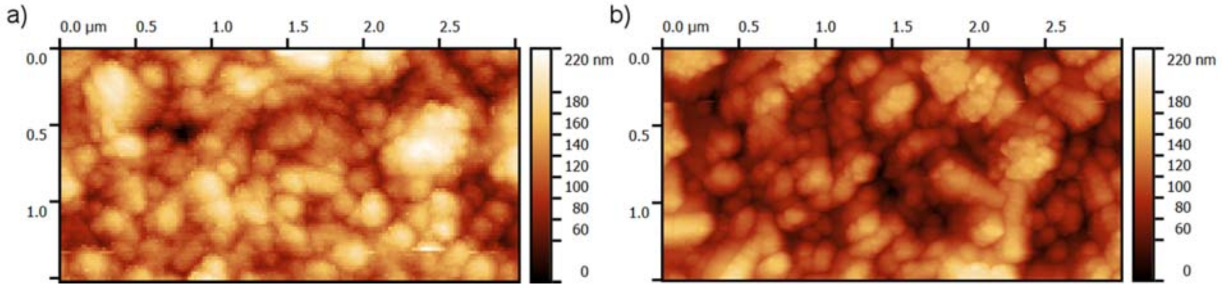

Figure S1. *Surface of a $Ni_{1-y-z}Fe_yCr_zO_x$ materials library recorded by AFM for a) low Cr content (50 at. % Ni, 35 at. % Fe, 15 at. % Cr) and b) high Cr content (48 at. % Ni, 12 at. % Fe, 40 at. % Cr).*

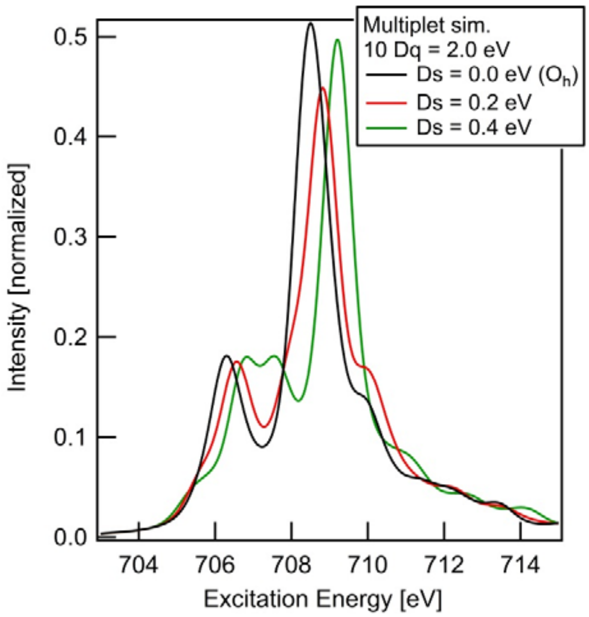

Figure S2. Multiplet simulation of Fe^{III} in D_{4h}/O_h symmetry performed with the software CTM4XAS[33]. The ligand field splitting 10 Dq is 2.0 eV in all cases and a distortion of the octahedral symmetry (D_{4h} symmetry) was modeled with a non-zero Ds value as given in the figure. No normalization was performed.

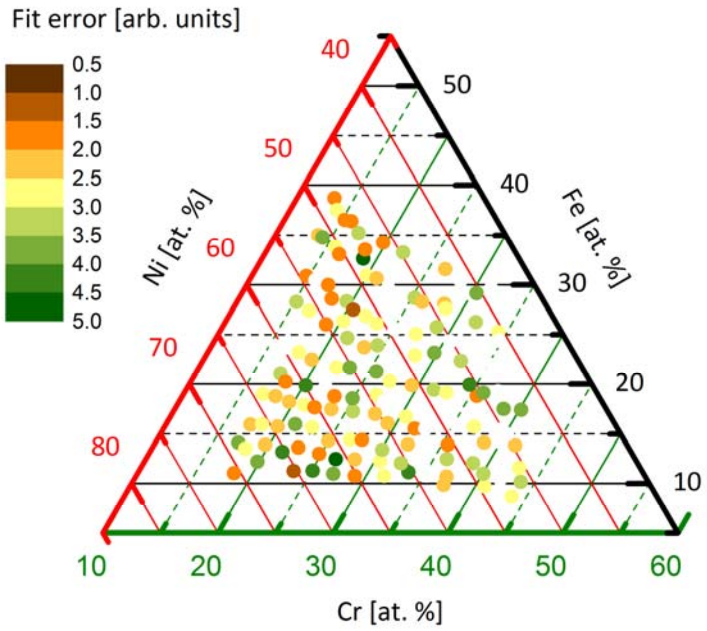

Figure S3. Error of the linear combination fit $\boldsymbol{\varepsilon}$ of the Fe L_3-edge with the two references Fe^{III} O_h and Fe^{III} T_d as shown in Figure 6 in the main text. $\boldsymbol{\varepsilon}$ is calculated as $\boldsymbol{\varepsilon} = \int \boldsymbol{abs}(\boldsymbol{S} - \boldsymbol{c_0}\boldsymbol{K_0} - \boldsymbol{c_1}\boldsymbol{K_1})\boldsymbol{dE}$, where $\boldsymbol{S}$ is the spectrum to be fitted, $\boldsymbol{c_0}$ and $\boldsymbol{c_1}$ are the coefficients from the linear combination fit for the Fe^{III} O_h and Fe^{III} T_d spectrum, respectively and $\boldsymbol{K_1}$ and $\boldsymbol{K_0}$ are the Fe^{III} O_h and Fe^{III} T_d spectra, respectively. The integral is performed over the excitation energy $\boldsymbol{E}$ at the L_3-edge.

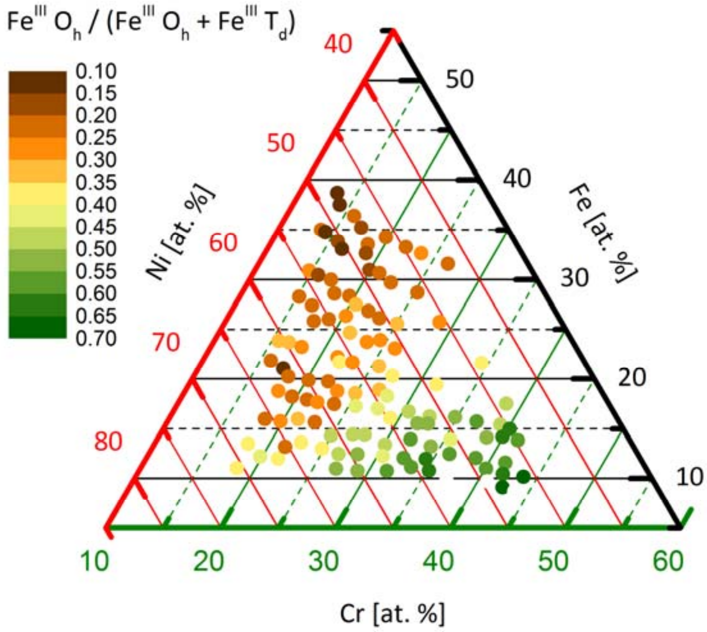

Figure S4. Ratio of the amounts of the two Fe species Fe^{III} O$_h$ and Fe^{III} T$_d$. Fe^{III} O$_h$ and Fe^{III} T$_d$ are shown in Figure 5. The same quantity is shown versus the Cr content in Figure 6.
